# Biotechnological Production of Carotenoids Using Low Cost-Substrates Is Influenced by Cultivation Parameters: A Review

**DOI:** 10.3390/ijms22168819

**Published:** 2021-08-17

**Authors:** Willen Silva Igreja, Felipe de Andrade Maia, Alessandra Santos Lopes, Renan Campos Chisté

**Affiliations:** 1 Graduate Program in Food Science and Technology (PPGCTA), Institute of Technology (ITEC), Federal University of Pará (UFPA), Belém 66075-110, PA, Brazil; willenchurch@gmail.com (W.S.I.); alessalopes@ufpa.br (A.S.L.); 2 Faculty of Food Engineering (FEA), Institute of Technology (ITEC), Federal University of Pará (UFPA), Belém 66075-110, PA, Brazil; fmaia780@gmail.com

**Keywords:** yeast, bioactive compounds, β-carotene, torulene, torularhodin, natural pigments

## Abstract

Carotenoids are natural lipophilic pigments mainly found in plants, but also found in some animals and can be synthesized by fungi, some bacteria, algae, and aphids. These pigments are used in food industries as natural replacements for artificial colors. Carotenoids are also known for their benefits to human health as antioxidants and some compounds have provitamin A activity. The production of carotenoids by biotechnological approaches might exceed yields obtained by extraction from plants or chemical synthesis. Many microorganisms are carotenoid producers; however, not all are industrially feasible. Therefore, in this review, we provide an overview regarding fungi that are potentially interesting to industry because of their capacity to produce carotenoids in response to stresses on the cultivation medium, focusing on low-cost substrates.

## 1. Introduction

Carotenoids are bioactive compounds found in plants, animals, and microorganisms. They are lipid-soluble and are used as food colorants (β-carotene, lycopene, lutein, astaxanthin, and bixin). Some specific compounds have provitamin A activity, and their consumption has been associated with strengthening the immunological system and reducing the risk of chronic degenerative diseases such as cancer, cardiovascular diseases, macular degeneration, cataracts, inflammation, and others [1].

Despite carotenoids being widely distributed in plants, their cellular contents are low, requiring large areas of cultivation to reach high production, specific agricultural practices, geographical variations, seasonality, and costs of the raw material and sometimes also requires manual harvest, justifying the high cost of these molecules [2]. Commercial production of carotenoids is carried out primarily by chemical synthesis or by extracting plants or algae. Many microorganisms biosynthesize carotenoids; however, not all are industrially or economically feasible. Products obtained by microbial pathways can be obtained quickly and at any time of the year and they have the advantages of low environmental pollution, low production costs when compared to the extraction from plants, and a high yield in addition to broad development prospects [3]. Over the years, several studies investigated the potential of carotenoid production by microorganisms such as yeasts through fermentation using various agricultural products and by-products [4,5,6,7,8,9].

Chemically synthetized carotenoids are less expensive; for example, astaxanthin has a lower production cost (≈$1000/kg) [10] when comparing to astaxanthin produced from *Haematococcus pluvialis* ($2500–7000/kg) [11], while β-carotene production by chemical synthesis is expected to be even less expensive since the theoretical operating costs to produce β-carotene from *Dunaliella salina* may vary from $343.54–499.59 [12]. However, on account of the growing concern about using chemical additives in food, there has been significant interest in carotenoids obtained naturally by biotechnological processes. 

According to Mantzouridou [13], the manufacturing and commercialization costs of the final product and its intended utilization influence the bioprocess viability. The production of carotenoids by fermentation might become industrially feasible if low-cost agroindustrial by-products can minimize their costs as sources of nutrients and make this process more environmentally friendly. However, the biotechnological processes to produce high yields of carotenoids are influenced by substrate composition, physicochemical characteristics of growing medium (pH, temperature), and the specific conditions of the process (agitation, aeration rate, and light exposure), all of which affect cellular growth and carotenoid profiles [8,14,15].

In this review, we summarized information on the production of the primary carotenoids produced by fungi (β-carotene, torulene, and torularhodin), their chemical structures, classification, properties, biosynthesis, producing microorganisms, conditions that control their bioproduction, and commercial use in Brazil and internationally.

## 2. Carotenoids: Chemical Structures, Classification, and Biosynthesis

Carotenoids are natural pigments synthesized by plants, bacteria, algae, fungi, and some arthropods such as hemipteran (aphids, adelgids, phylloxerids) [16]; humans and other animals must obtain these compounds from food sources [17]. More than 750 carotenoids have been reported. They are primarily yellow, orange, and red; however, there are also colorless carotenoids, including phytoene and phytofluene [18,19,20,21].

The chemical structures of carotenoids include isoprenoids and forty-carbon tetraterpenes formed by eight units of isoprenes and an extensive system of conjugated double bonds that generate electron resonance systems (π) moving throughout the entire polyene chain. Owing to these structural characteristics, carotenoids are highly reactive molecules and mostly absorb electromagnetic radiation in the visible region (360–780 nm) [22]. This basic structure can be altered by chemical reactions such as hydrogenation, dehydrogenation, cyclization, double bond migration, chain shortening or extension, rearrangement, isomerization, the introduction of substitutes, and oxidation, all of which influence the chemical, physical, and biochemical properties [23,24,25,26].

**Figure 1:**
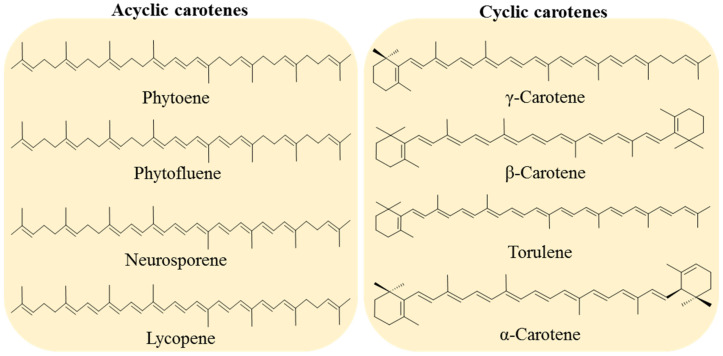
Examples of acyclic carotenes, all containing two linear ψ-end groups: phytoene, phytofluene, neurosporene, and lycopene. Cyclic carotenes with one or two β-rings (γ-carotene, β-carotene and torulene) and one β- and one ε-rings (α-carotene).

**Figure 2:**
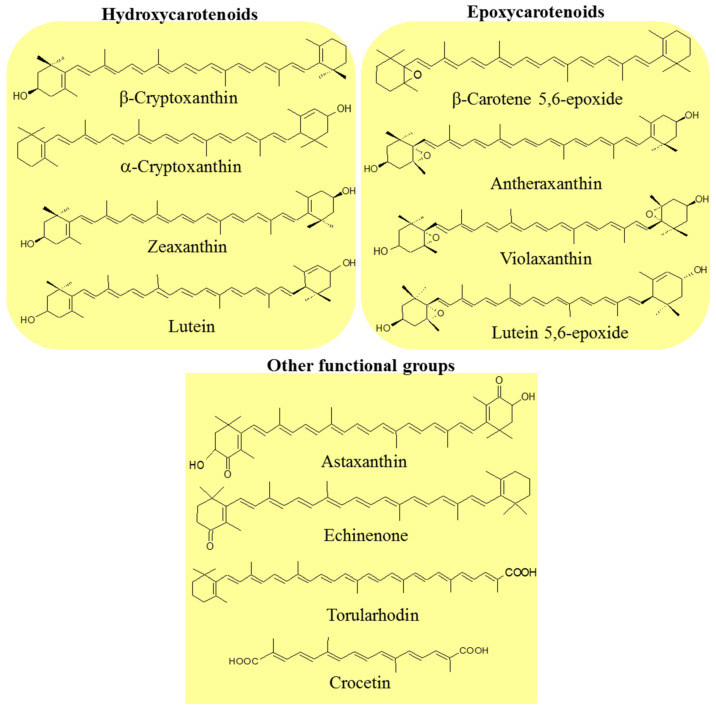
Examples of xanthophylls with some oxygenated functional groups in their structures: hydroxyl (hydroxycarotenoids), epoxy (expoxycarotenoids), ketone (astaxanthin and echinenone), and carboxylic acid (torularhodin and crocetin).

Carotenoids are classified into two groups: carotenes, formed by compounds with carbon and hydrogen (hydrocarbons) in their chemical structure, and xanthophylls, which are the oxygenated derivatives of carotenes. Carotenes can be found as linear hydrocarbons which can be cyclized with specific end-groups in one or both sides of the molecule (Figure 1), while xanthophylls, which are the oxygenated derivatives compounds, may contain different functional groups such as hydroxyl, ketone, carboxylic acid, and epoxy (Figure 2) [21].

Various microorganisms can biosynthesize carotenoids; however, many are not commercially feasible. Yeasts, in particular, grow on low-cost substrates such as *Phaffia rhodozyme* [27,28,29], *Sporobolomyces* [30,31], *Rhodosporidium*, and *Rhodotorula* [32,33,34]. In these species, β-carotene, astaxanthin, γ-carotene, torulene, and torularhodin are the major carotenoids (Figure 1 and Figure 2).

**Figure 3:**
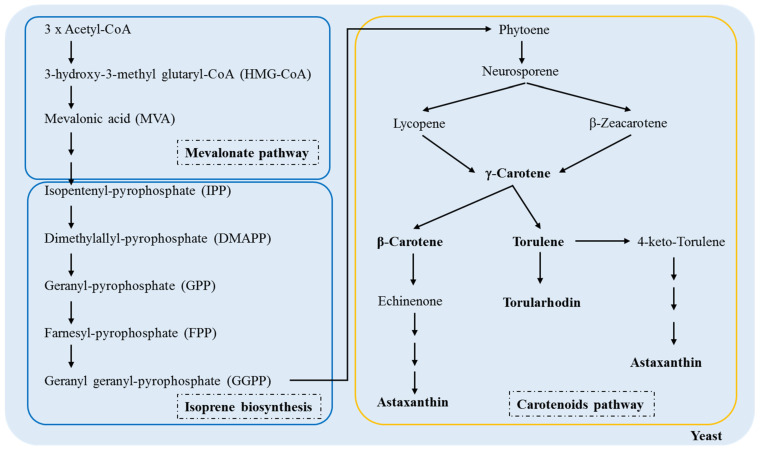
Biosynthetic pathway of the carotenoid production in yeasts, adapted from Frengova & Beshkova [38].

Knowledge of the molecular mechanisms that produce carotenoids can help improve biotechnological processes; therefore, their biosynthesis in fungi has been an object of investigation for many years [35,36,37]. Figure 3 shows a schematic of the carotenoid pathway in yeast from acetyl-CoA.

There is a conversion of three molecules of acetyl-CoA at 3-hydroxy-3-methylglutaryl-CoA (HMG-CoA) by an enzyme in the mevalonic acid pathway HMG-CoA synthase. HMG-CoA is catabolized by 3-hydroxy-3-methylglutaryl-CoA reductase, transforming into a six-carbon compound called mevalonic acid (MVA), the first precursor of the biosynthetic pathway of terpenoids. Then, MVA is phosphorylated, pyrophosphorylated, decarboxylated, and dehydrated to produce isopentenyl-pyrophosphate (IPP), the basic unit of the formation of terpenes by quinase and decarboxylase [38,39].

In the first stage of isoprene biosynthesis in yeasts, IPP and DMAPP molecules react to form a larger compound called geranyl-pyrophosphate, a ten-carbon molecule. Then, there is the addition of IPP sequences in the GP molecule to form farnesyl-pyrophosphate (FPP) (15 carbons), the precursor of most sesquiterpenes. Later, another IPP molecule is added to the FPP molecule to form geranyl geranyl-pyrophosphate (GGPP), the precursor of diterpenes composed of 20 carbons. Prenyl transferase catalyzes these reactions. Finally, FPP and GGPP dimerize to form triterpenes (30 carbons) and tetraterpenes (40 carbons), respectively [38,39,40].

The condensation of the two molecules of GGPP leads to the formation of phytoene, the first 40-carbon carotenoid; in turn, these are desaturated to form phytofluene, neurosporene and lycopene [41,42]. Because lycopene is a *trans* compound, isomerization of the first or second double bond of phytoene occurs in the same stage as the desaturation reaction [43]. Lycopene is a precursor of the cyclic carotenoids, and its cyclization leads to the formation of γ-carotene, β-carotene, echinenone, torulene, torularhodin, and astaxanthin [44,45]. In the carotenoid pathway in yeasts, γ-carotene appears to be the critical point in the ramification because it acts as the precursor of β-carotene and torulene. In turn, hydroxylation and oxidation of torulene lead to the formation of torularhodin [42].

## 3. Main Carotenoids Produced by Fungi

**Figure 4:**
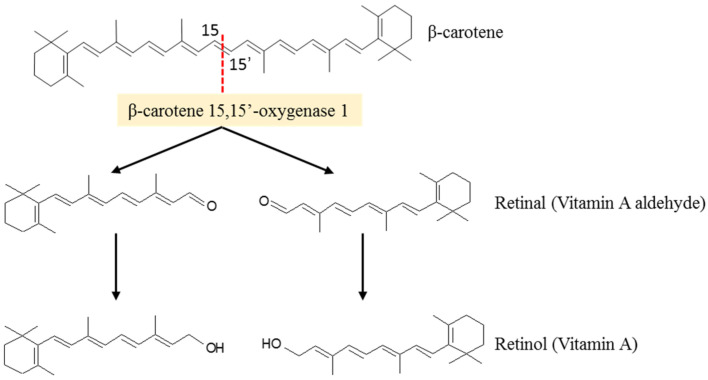
Enzymatic cleavage of β-carotene produces two molecules of retinol (vitamin A) (adapted from [48]).

According to several studies, the carotenoid biosynthesize in fungi is stimulated as a response to the stresses introduced by the growth medium. Among the more than 750 known carotenoids, about 50 can be metabolized in vitamin A [21,46]. Among them, β-carotene is the most relevant because of its high provitamin A activity. For vitamin A activity, a carotenoid must have at least one unsubstituted β-ionone ring with an attached polyene side chain of at least eleven carbons [47]. Because β-carotene has 40 carbon atoms and two unsubstituted-β-rings with attached polyene side chains of eleven carbons, it may undergo enzymatic cleavage in the small intestine, liver, adipocytes and adipose tissues, at the center of the polyene chain, mediated by β-carotene 15,15′-oxygenase 1, into two retinal molecules to be further converted into retinol (Vitamin A) (Figure 4) [48,49].

The microbiological production of β-carotene occurs in microorganisms such as *Blakeslea trispora* [50], *Phaffia rhodozyma* [27], *Rhodosporidium toruloides* [51], *Sporidiobolus salmonicolor* [14], and *Rhodotorula* spp. [34,36,52] (Table 1). β-carotene has remarkable antioxidant properties by inhibiting or delaying oxidative dam-age both in physiological and food systems [53,54,55,56].

Microorganisms frequently produce another carotenoid of interest, γ-carotene (Figure 1). Owing to the 11 conjugated double bonds and the presence of one unsubstituted β-ionone ring attached to a polyene side chain in its chemical structure, γ-carotene has the structural requirement to be converted into one retinol molecule, but such bioconversion has not been well-characterized. The biotechnological production of γ-carotene through yeast was reported in several strains of *Rhodotorula* and *Sporobolomyces* [30,36]. Although γ-carotene can be found in fruits [58], it is less common than β-carotene, and there are few studies about its chemical properties, bioavailability, and antioxidant capacity [31,59], and it can be used as a food colorant [47].

Torulene is a carotenoid synthesized mainly by microorganisms (Figure 1). Its chemical structure possesses 40 carbons and one unsubstituted β-ionone ring attached to a polyene side chain of 11 carbons. Torulene possesses 50% of the provitamin A activity of β-carotene. This natural red pigment was reported as the major carotenoid produced by R. mucilaginosa [30], and it has the potential to be produced by biotechnological means, similar to food colorants such as β-carotene, or lycopene, in addition to its provitamin A activity and antioxidant capacity [44,65].

Torularhodin is a xanthophyll derived from torulene; it possesses 40 carbons and a β-ionone ring but with a carboxylic acid group in its structure [44,66] (Figure 2). As a colorant, these characteristics can be advantageous in specific food formulations because the presence of carboxyl group increases its solubility in aqueous formulations, allowing the same treatment used for bixin and norbixin in meat products [44,65], along with its high antioxidant capacity [66,67]. Many microorganisms can biosynthesize torulene and torularhodin, including *Rhodotorula rubra* [68] and *Neurospora crassa* [69,70].

Another xanthophyll produced by microorganisms, astaxanthin (Figure 2), has 40 carbons, a long polyene chain with 13 conjugated double bonds and two substituted β-rings with both hydroxyl and ketone functional groups. Therefore, it does not possess provitamin A activity. Astaxanthin is responsible for the characteristic color of fish such as trout and salmon, crustaceans, and birds; however, it is also used as a colorant in some foodstuffs and cosmetics. Following β-carotene, astaxanthin is the second most commercially vital carotenoid, representing about 26% of the total sales of carotenoids [71]. The market for carotenoids reached $288.7 million in 2017 and should reach $426.9 million by 2022 [72]. The biotechnological production of astaxanthin primarily involves the yeast *Xanthophyllomyces dendrorhous* (anamorph *Phaffia rhodozyma*) [73].

In yeasts, the major carotenoids biosynthesized by four strains of *Rhodotorula* are torulene and β-carotene, followed by γ-carotene and torularhodin, varying about 103–250 μg of total carotenoids/g of freeze-dried biomass [36]. Using low-cost substrates, Banzatto et al. [74] reported that *R. rubra* cultivated in molasses produced substantial amounts of carotenoids (~329 µg/g, dry basis) with no need for additional nutrient supplementation. The major carotenoids were torulene, torularhodin, and β-carotene. In another study with *R. mucilaginosa*, molasses was a promising source for the low-cost production of torulene, β-carotene, and torularhodin [34].

In a study investigating the production of microbial fats for the production of biodiesel and high-value carotenoids by *R. glutinis*, brewery effluents as carbon sources allowed the production of carotenoids in all treatments (from 0.6 to 1.2 µg/mL) with high proportions of β-carotene (∼50%) [75]. Zhang et al. [37] measured the capacity of *R. glutinis* to produce fats and carotenoids under various conditions of irradiation, temperature, and carbon/nitrogen ratios. They found that low temperature/dark environment increased the fatty content, while irradiation/high temperature increased the production of biomass and carotenoids. In another study, the relations among the production of carotenoids, copper bioremediation, and oxidative stress of *R. mucilaginosa* RCL-11 were assessed [76]. The authors observed changes in the proportions of torularhodin, torulene, and β-carotene that depended on the stresses applied to the yeasts.

Cardoso et al. [31] investigated the improvement in the production of carotenoids by *Sporobolomyces ruberrimus* using raw glycerol and identified the presence of torularhodin, torulene, β-carotene, and γ-carotene. These authors concluded that raw glycerol increased the proportion of torularhodin, and the addition of individual fatty acids in pure glycerol resulted in an increase in the productivity of carotenoids by 15–25%. In another study, Varmira et al. [77] investigated the effect of mineral salts and solvents in carotenogenesis by *R. rubra*, using glucose as a carbon source and ammonium sulfate as a nitrogen source. These authors demonstrated that carotenogenesis improved in magnesium sulfate (MgSO4), and its combination with methanol had a more significant impact on the carotenogenesis performance of the cell torularhodin, torulene, and β-carotene, the main carotenoids.

## 4. Antioxidant Potential of Torulene and Torularhodin

In addition to their potential provitamin A activity, β-carotene, torulene, and torularhodin (the most frequently studied carotenoids produced by fungi) also have high antioxidant capacity [41,44], as demonstrated in some studies. The antioxidant properties of β-carotene have been investigated repeatedly, as reviewed elsewhere [78,79]. Because torulene and torularhodin are not components of human diets because of their absence in vegetable and animal food sources, they are not yet industrially produced, and their influence on human health is not yet apparent [41]. Nevertheless, based on their chemical structures and claimed properties, these carotenoids are promising compounds as additives in food, cosmetics formulations [44], and even drug ingredients [68].

Torulene is the primary carotenoid produced by yeasts from genus *Rhodotorula*. This carotenoid has potential for industrial applications [44], while torularhodin is one of the few carotenoids with a carboxylic acid in its chemical structure; it showed in vitro antioxidant activity against singlet oxygen and peroxyl radicals [67]. Sakaki et al. [80] reported that torularhodin and torulene were more efficient singlet oxygen quenchers than β-carotene due to their higher number of conjugated double bonds Interestingly, the same behavior was also observed for torularhodin and torulene against peroxyl radicals, and they presented higher scavenging capacity than β-carotene [67]. These observations support studies regarding the inhibition or delay of lipid peroxidation in both food and physiological systems.

The oxidative damage in the stroma cells in the human prostate induced by hydrogen peroxide (H_2_O_2_) was treated with the antioxidants torulene and torularhodin to prevent the onset and progression of prostate diseases [81]. These authors showed that both carotenoids protected stroma cells against oxidative damage mediated by the overproduction of reactive oxygen species via regulation of Bcl-2/Bax-mediated apoptosis, with activity higher than that of lycopene.

## 5. Important Industrial Aspects of Carotenoid Production by Biotechnological Approaches

Industrial production of food colorants has been expanding in recent decades; however, due to the constant search for natural products aiming to overcome any concern about health by modern consumers, the international pigment market tried to stimulate the use of natural pigments in their products instead of the synthetic or artificial ones.

In 2019, the carotenoids market in the world reached 1.5 billion dollars and the prediction is to reach 2.0 billion dollars in 2026, which highlights it as a promising market with a number of opportunities in business [13].

In Brazil, trade data on the import and export of carotenoids can be found in the Brazilian foreign trade statistics data portal [82] by entering the standard nomenclature of Mercosul (NCM) of the selected products. For carotenoid-related products, three searches are made possible by NCM 32041911 (carotenoids), NCM 32041912 (preparations containing beta-carotene, methyl or ethyl esters of the 8′-apo-beta-carotene acid or canthaxanthin, with vegetable oils or fats, starch, gelatin, sucrose or dextrin, proper to stain food), and NCM 32041919 (other carotenoid-based preparations). The values (USD) and the imported and exported quantities (kg) of these three NCM are displayed in Table 2.

From 2013 to 2018, Brazil imported a total of 3,781,376 kg of the three NCMs mentioned above, costing $38,929,505. In the same period, Brazil exported only 10,555 kg, corresponding to $678,007, making Brazil a net importer of carotenoids and their derivatives. The global carotenoid market is evaluated at an annual rate of 2.3% transacting $1.4 billion as of 2018 [83]. Approximately 90% of carotenoids on the market are derived from chemical synthesis; however, because of the growing concern about the use of chemical additives in food, the market for colorants produced by chemical synthesis has been limited to satisfy consumer desire for natural pigments [84]. In this sense, pigments synthesized by fungi and other microorganisms have been attracting interest from the scientific and commercial communities.

The possibility of natural pigment production on the industrial scale and the increased aggregate value of the products makes biotechnological production of carotenoids an area of intensive research. These pigments can be produced by industrial fermentation, where there is a growth phase to increase the microbial biomass followed by a production phase, in which the biomass remains constant; however, the carotenoid synthesis is increased [17]. The search for natural pigments drove various countries to invest in natural carotenoid production using biotechnological pathways (Table 3).

## 6. Factors That Affect Biotechnological Production of Carotenoids

The productivity of a biotechnological process in any given system depends on the nutritional and physical conditions of the culture that affect cellular growth and pigment production. The evidence suggests that optimum conditions for carotenoid production are not the same as those of cell growth because carotenoid biosynthesis and differences in carotenoid profiles and amounts can be influenced in response to environmental stress conditions [8,14,15].

Therefore, knowledge of cultivation conditions such as cultivation temperature, aeration, pH, lighting, and composition of the substrates is of paramount importance to obtain processes that stimulate microorganisms to modulate carotenoid production and composition of interest; and they were summarized in Table 4 and Table 5. Furthermore, assessing all these factors is essential for the industrial development of carotenoid production by biotechnological approaches. To be industrially feasible, it is necessary to reduce production costs, improve carotenoid yields, and investigate techniques to improve recovery (extraction and isolation) and preserve these compounds.

### 6.1. pH

pH is a significant environmental parameter that influences carotenogenesis by modulating cell growth and biosynthesis of carotenoids [15]. During the biosynthesis of carotenoids in fermentation processes, there is a natural change of pH in the cultivation medium according to the yeast growth. In general, pH decreases during the first 72 h, followed by increased pH values due to an intensive carotenogenesis phase; at the end of the bioproduction of carotenoids, pH values remain constant [96].

Tinoi et al. [5] studied the optimum conditions to produce carotenoids by the yeast *R. glutinis*. They used a substrate containing hydrolyzed mung bean waste flour from glass noodle production as the principal nitrogen source and sweet potato extract as the principal carbon source at pH 3.0–7.5. They reported that the highest total carotenoid contents (3.48 µg/mL) and biomass production (10.35 g/L) were achieved at the optimum pH of 5.91.

The optimum pH value for a strain of yeast *R. glutinis* isolated from the sweet scabious flower (*Scabiosa atropurpura*) was reported at pH 6.2 [35]. The authors of this study cultivated the yeast strain in synthetic medium (zinc sulfate (0.1 g/L) and sucrose (12.5 g/L) at 25 °C, for six days, and reported that most of the carotenoid contents were produced during the stationary phase, with the highest content of total carotenoids (861 μg/g) observed after five days of growth. In a study of fermentation in solid state (culture medium based on YM broth contains imidazole as an inducer of production of lycopene), the optimum cultivation conditions for *R. glutinis* included pH values close to 4.0, with total carotenoids’ values of 340 μg/mL [86]. In another study, Varmira et al. [77] reported pH 5.0 as an optimum value for both the production of biomass and carotenoids by *R. rubra* and that pH decreased to 2.0 inhibited the yeast growth completely. That study used a culture medium in the presence of mineral salts (at 0.1 mg/L for FeSO_4_, CaCl_2_, and MgSO_4_) and solvents (2% vv-1 of ethanol and methanol), using glucose as a carbon source and ammonium sulfate as a nitrogen source.

Naghavi et al. [87] investigated the influence of pH (3–8) in *R. mucilaginosa* using synthetic culture medium containing glucose (NH_4_)_2_SO_4_, KH_2_(PO_4_), MgSO_4_, CaCl_2_, and yeast extract. The authors observed that an increase of pH to 5 had a significant effect on the production of carotenoids and biomass; however, higher pH values caused significant decreases in both factors. The highest dry biomass and total carotenoid contents were 16.33 g/L and 3930 μg/mL, respectively.

For the same yeast (*R. mucilaginona*), another study reported that an increase of pH from 3.0 to 7.0 increased the cell growth (5.1 g/L dry cells) and total carotenoid contents (69.8 μg/mL); moreover, the authors used glucose, molasses sucrose, and whey lactose sugars as carbon sources. In general, the increase in sugar concentration increased the growth of yeast and total carotenoid production. The highest carotenoid concentration (89.0 mg total carotenoids per liter of fermentation broth) was obtained when 20 g/L molasses sucrose was used as the carbon source, while the highest product yield (35.0 mg total carotenoids per gram of dry cells) was achieved when whey lactose (13.2 g/L) was the carbon source [52].

Nasrabadi & Razavi [88] reported that the optimum conditions to produce β-carotene for mutant yeast *R. acheniorum* at pH 5.85 resulted in a 4.62-fold increase in accumulation of β-carotene (262.12 μg/g). For *Sporidiobolus salmonicolor* (CBS 2636), the bioproduction of carotenoids was partially associated with cell growth, and the maximum concentration of total carotenoids (3.42 μg/mL) was achieved in a bioreactor with initial pH of 4.0 [93]. In another study, *Rhodosporidium diobovatum* yeasts were inoculated at an initial pH value of 5.5, and a decrease in the pH value (4.2) was observed after 96 h of incubation, followed by a pH peak at 5.0 in 120 h of incubation; these findings were highlighted by the highest total carotenoid contents (186 μg/g) [89].

Shih & Hang [61] assessed three strains of *R. rubra* in an acidic medium and observed that, at low pH values (pH 3.4–4.5), there was inhibition of the cell growth and the production of carotenoids, considering that the maximum cell concentration and pigment production was obtained at initial pH = 5.0. In another study, Mihalcea et al. [90] studied the effect of pH in a range from 3.0 to 8.0 in *R. rubra* and showed that the optimum pH for the yeast growth was pH 5.0. These authors also showed that the formation of torularhodin was favored in the pH range 6.0–7.0; at pH 8.0, cell growth was limited, and at pH 3, the production of carotenoids was not favorable.

These findings suggest that pH highly influences carotenogenesis in yeasts. In general, these microorganisms prefer more acidic pH values; however, at very low pH values, there is inhibition of yeast growth and consequent reduction of carotenoid production.

### 6.2. Temperature

The average fungus growth temperature is 25°–30 °C. According to Valduga et al. [14], the temperature is one of the most critical environmental factors that influence the growth and development of microorganisms. Temperature affects many biosynthetic pathways, including carotenogenesis. Regarding carotenogenesis, temperature influences the control of enzyme concentrations, and any variations in these concentrations can modulate the biotechnological production of carotenoids [97].

Contrasting results concerning the effect of temperature on carotenoid production by fungi can be found in the literature. El-Banna et al. [35] reported that the optimum temperature to produce carotenoids by *R. glutinis* was 15 °C, while cell growth (dry biomass) was produced in more significant quantities at 25 °C. These authors also highlighted the influence of cultivation temperature on carotenoid profile; the higher the temperature, the greater the β-carotene contents and the lower torulene and torularhodin contents. In another set of experiments with *R. glutinis* subjected to various irradiation conditions, temperatures and carbon/nitrogen ratios, low temperatures (24 °C), and dark environment favored fatty contents. In contrast, high luminosity combined with higher temperature (30 °C) increased the production of biomass and carotenoids [37].

Aksun and Eren [6] assessed the specific growth rate of *R. glutinis* cells and concluded that the growth rate increased with increased temperature (25 °C to 30 °C) and reduced drastically at a higher temperature. By contrast, these authors observed that the production rate of carotenoids increased at temperatures above 30 °C. For *R. mucilaginosa*, higher temperature (25 °C to 30 °C) increased the carotenoid production rate; however, at temperatures above 30 °C, carotenoid biosynthesis appeared to be reduced, and this finding was associated with the denaturation of the yeast enzymatic system [52]. In another study with *R. mucilaginosa*, both biomass yield and carotenoid contents increased with temperature increase (from 10 to 30 °C) [87].

For mutant *R. acheniorum* isolated from milk whey, the maximum optimized production of β-carotene (262.12 μg/mL) was reported at 23 °C [88]. However, *R. rubra* did not show significant difference at temperatures within 20–30 °C for both production of carotenoids and biomass growth [90], while inoculation temperature at 30 °C provided maximum cell growth for *R. diobovatum* [89], and the highest production of carotenoid was found at 25 °C for *Sporidiobolus salmonicolor* [42].

Generally, temperature ranges that are ideal for carotenogenesis are not the same as those of cell growth; hence, high temperature ranges should be approached during both processes. However, low temperature usually does not contribute to cell growth and does not contribute to carotenoid production. On the other hand, high temperatures may denature enzymes necessary for carotenogenesis and may inhibit cell growth.

### 6.3. Agitation and Aeration Rate

Aerobic microorganisms require aeration and agitation conditions to achieve higher yields. Tinoi et al. [5], using hydrolyzed mung bean waste flour as substrate, demonstrated that lower cell growth of *R. glutinis* occurred at low agitation rates (100 to 150 rpm) due to the reduction of the availability of nutrients on the cell surface; however, cell rupture was observed at high agitation rates (>250 rpm). For carotenoid production by *R. glutinis*, El-Banna et al. [35] reported the highest carotenoid contents (1.9 μg/mL) after cultivation at 25 °C during constant agitation at 100 rpm.

In another study with *R. glutinis*, the cultivation conditions with and without agitation were studied, and the highest total carotenoid concentrations were obtained after agitation at 125 rpm during the fermentation process [86]. For *R. mucilaginosa*, incubation in a rotary agitator at 150 rpm for 72 h resulted in maximized carotenoid production (3.40 μg/mL) [87]. Liu et al. [27] demonstrated the strong influence of external oxygen transfer provided by agitation on *P. rhodozyma* growth and carotenoid production in liquid cultures because the coefficient of oxygen transfer increases with agitation rate. These authors suggested that carotenoid biosynthesis may be enhanced by increasing the respiration activity of these cells.

The limitation of oxygen negatively affected biomass production and, consequently, the concentration of total carotenoids by *Rhodosporidium toruloides* (NCYC 921) using carob pulp syrup as a substrate [91]. Valduga et al. [42] reported an aeration rate of 1.5 volumes of air per volume of medium per minute (vvm) and 180 rpm to maximize the production of carotenoids by *Sporidiobolus salmonicolor* (CBS 2636). The importance of the aeration process during fermentation in various agroindustrial media was also demonstrated by Borba et al. [95]. They produced carotenoids by *Sporidiobolus pararoseus*, which was 3.5-fold higher (1969 µg/L) in a stirred tank (158 rpm and 1.2 vvm) than the value found during agitation in shaking flasks (100–200 rpm). These authors demonstrate the influence of agitation on the production of carotenoids by *S. pararoseus* and the effect of temperature and pH. The combined investigated conditions allowed them to conclude that carotenoid production capacity can be increased by varying the aeration and agitation parameters.

### 6.4. Light Irradiation

Carotenogenesis in algae, fungi, and bacteria is positively affected by white light irradiation [14]; production and accumulation of carotenoids depend on light intensity illumination and the type of microorganism. Bhosale [98] described the photo-induction theory in two phases: the first is related to the effect of stimulation of production the white light can induce on microbial growth; the second phase considers that carotenoid accumulation in the cell is associated with the increase in the activity of the enzymes involved in carotenogenesis.

Sakaki et al. [66] investigated the production of torularhodin by *R. glutinis* using weak white light irradiation, which inhibited cell growth; however, simultaneously, it showed a substantial increase in carotenoid production. These authors also demonstrated that such inhibition of cell growth depended on the type of microorganism because, at the same light irradiation condition, no effect on the cell growth was observed for *Saccharomyces cerevisiae*. These findings suggested that selected yeasts can biosynthesize carotenoids to respond to possible cell damage induced by light exposure.

The light produced by light-emitting diodes (LEDs) appeared to affect carotenoid accumulation in *R. glutinis*. A content of 2.6 µg carotenoids/mL was obtained when the yeasts were subjected to illumination by LED lamps (800 mol/m^2^s), and this value increased two-fold as to the control culture with *Haematococcus pluvialis* without illumination when the culture was illuminated with three LED lamps. The authors also reported that the light incidence (three LED lamps with 800 mol/m^2^s of one LED lamp) did not inhibit the yeast growth [37].

### 6.5. Substrate Composition

The type and composition of the substrate directly impact the yield of pigments, and, consequently, the cost of biotechnological processes; substrates composed of sucrose and glucose were the most reported carbon sources in the bioproduction of carotenoids [14]. Marova et al. [99] stated that the best conditions to achieve maximum yield of carotenoids is maintaining high cell growth rates and availability of carbon sources. Therefore, various by-products and raw materials from food industries or agroindustries have been investigated as promising substrates for microorganism growth and carotenoid production (Table 5) because of their high nutrient availability and low acquisition cost, and the feasibility of the industrial biotechnological processes.

**Table 5:** Agroindustrial residues investigated as substrates for carotenoids production by yeasts.

Species	Substrate	Carotenoid	Reference
*Rhodotorula* * glutinis*	Soybean extract	β-carotene, torulene, torularhodin	[4]
*Rhodotorula* * glutinis*	Glucose syrup	β-carotene, torulene, torularhodin	[4]
*Rhodotorula* * glutinis*	Ultra-filtered whey	Total carotenoids	[96,100]
*Rhodotorula* * glutinis*	Corn extract	β-carotene, torulene, torularhodin	[4]
*Rhodotorula* * glutinis*	Raw glycerol	Total carotenoids	[101]
*Rhodotorula* * glutinis*	Mung bean flour and sweet potato extract	Total carotenoids	[5]
*Rhodotorula* * glutinis*	Whey	β-carotene	[99]
*Rhodotorula* * glutinis*	Fermented radish brine	β-carotene	[102]
*Rhodotorula* * glutinis*	Chicken feathers	Total carotenoids	[103]
*Rhodotorula* * glutinis*	brewery effluents	β-carotene	[75]
*Rhodotorula* * glutinis*	Residual effluent from potato starch	Torularhodin, torulene and β-carotene	[104]
*Rhodotorula* * glutinis*	Beetroot molasses	β-carotene, torulene, torularhodin	[4]
*Rhodotorula* * glutinis*	Grapes must	β-carotene, torulene, torularhodin	[4]
*Rhodotorula* * rubra*	Media based on sugarcane broth, molasses and syrup	Torulene, torularhodin and β-carotene	[74]
*Rhodotorula* * rubra*	Ultra-filtered milk whey	Torulene, torularhodin and β-carotene	[105]
*Rhodotorula* * rubra*	Sugarcane broth	Total carotenoids	[106]
*Rhodotorula* * mucilaginosa*	Potatoes	β-carotene	[99]
*Rhodotorula* * mucilaginosa*	Coffee residues	β-carotene	[107]
*Rhodotorula mucilaginosa*	Molasses	Torulene, torularhodin and β-carotene	[34]
*Rhodotorula mucilaginosa*	sisal bagasse hydrolyzate	Total carotenoids	[92]
*Rhodotorula mucilaginosa and Rhodotorula toruloides*	sugar beet pulp hydrolysates	Total carotenoids	[108]
*Rhodotorula* * acheniorum*	Ultra-filtered whey	β-carotene	[88]
*Sporidiobolus* * salmonicolor*	Corn wet-milling water	β-carotene	[107]
*Sporidiobolus* * salmonicolo*	Water from rice parboiling	β-carotene	[109]
*Sporidiobolus pararoseus*	corn steep liquor and pre-treated sugarcane molasses	Total carotenoids	[95]
*Rhodosporidium toruloides*	Carob pulp syrup	Total carotenoids	[91]
*Rhodosporidium toruloides*	*Camelina sativa* meal hydrolysates	Total carotenoids	[94]
*Phaffia rhodozyma*	Flower of *Calendula officinalis*, *Zea mays* seed flour, potato seed flour, *Pennisetum glaucum* seed flour, *Triticum* flour.	Astaxanthin	[29]

## 7. Conclusions and Future Perspectives

Biotechnological production of carotenoids by fungi is a promising industrial strategy because it enables agroindustrial residues as substrates in fermentation processes. These processes are less costly and contribute to environmental preservation. This review summarized information regarding strategies and biotechnological parameters that modulate the production of microbial carotenoids.

The application of carotenoids produced by microorganisms represents an expanding market that comprises poultry farming, aquafarming, supplements, cosmetics, animal food, pharmaceutical, and foods. As shown in the literature, the biological effects of the specific carotenoids produced by yeasts such as torulene and torularhodin, both in vivo and in vitro, have focused on studies of their use in the prevention of diseases and natural oxidative processes. However, there is a lack of literature describing definitive mechanisms by which these carotenoids may be used in physiological processes.

## Figures and Tables

**Table 1:** Main carotenoids produced by fungi by biotechnological approaches.

Species	Carotenoid	References
*Blakeslea trispora*	β-Carotene and lycopene	[50]
*Dacrymyces deliquescens*	Lutein	[57]
*Neurospora crassa*	Torularhodin, torulene	[58,59]
*Phaffia rhodozyma*	Astaxanthin and β-carotene	[27,28]
*Rhodosporidium* sp	Torulene, β-carotene	[57]
*Rhodosporidium toruloides*	β-Carotene	[51]
*Rhodotorula graminis*	Torulene	[60]
*Rhodotorula mucilaginosa*	Torularhodin, β-carotene, torulene	[4,52]
*Rhodotorula rubra*	β-Carotene, Torularhodin, torulene	[33,61]
*Rhodotorula glutinis*	Torularhodin, β-carotene, torulene	[4]
*Sporidiobolus salmonicolor*	β-Carotene	[42]
*Sporidiobolus* sp	Torularhodin, β-carotene, torulene	[57]
*Sporobolomyces roseus*	Torularhodin, β-carotene, torulene	[62]
*Sporobolomyces ruberrimus*	Torularhodin, β-Carotene	[63]
*Sporobolomyces ruberrimus*	Torularhodin, torulene, β-carotene and γ-carotene	[31]
*Saccharomyces cerevisiae*	β-Carotene	[64]

**Table 2:** Brazilian trade data related to the import and export of carotenoids, preparations containing β-carotene and other carotenoid-based preparations from 2013 to 2018.

	NCM 32041911		NCM 32041912		NCM 32041919	
	**Import** **(USD)**	** Export** **(USD)**	**Import** **(USD)**	**Export** **(USD)**	**Import** **(USD)**	**Export** **(USD)**
2013	61,476	573	104,752	1021	292,859	15
2014	62,184	800	97,330	805	284,614	62
2015	113,916	811	92,868	0	468,364	30
2016	79,062	2901	79,985	175	158,177	130
2017	118,259	846	58,578	190	926,866	31
2018	41,901	220	52,382	1180	687,803	765
	**Import** **(USD)**	**Export** **(USD)**	**Import** **(USD)**	**Export ** **(USD)**	**Import** **(USD)**	**Export** **(USD)**
2013	2,985,376	40,140	3,998,369	121,405	1,227,018	276
2014	2,985,827	50,512	2,771,384	52,834	991,527	1248
2015	3,809,948	60,281	2,730,419	36	1,366,252	521
2016	1,563,544	170,994	2,948,609	20,464	451,865	2552
2017	1,577,405	66,234	2,180,819	4492	2,051,999	612
2018	1,416,461	14,311	2,203,618	59,448	1,669,065	11,647

Source: Brazilian foreign trade statistics data portal [82]. NCM 32041911 = Brazilian carotenoid market; NCM 32041912 = preparations containing beta-carotene, methyl or ethyl esters of the 8’-apo-beta-carotene acid or canthaxanthin, with vegetable oils or fats, starch, gelatin, sucrose or dextrin, proper to stain food; and NCM 32041919 = other carotenoid-based preparations.

**Table 3:** Companies producing carotenoids by biotechnological pathway.

Company	Final Product	Country of Manufacture	Website
Alga Technologies	Oleoresin, capsules, powder and emulsion of astaxanthin	Israel	algatech.com
BASF	Mixture of carotenoids	Australia	worldaccount.basf.com
BlueBiotech	Micro-algae powder with astaxanthin	Germany	bluebiotech.de/com
Cyanotech	Astaxanthin capsules	USA	cyanotech.com
Fuji Chemical Industries	Astaxanthin in jelly capsules, tablets, powder and micro-algae biomass	USA and Sweden	astareal.com
Parry Nutraceuticals	Astaxanthin and mixture of β-carotene, zeaxanthin, cryptoxanthin and lutein	India	parrynutraceuticals.com
Plankton Australia Pty Limited	Micro-algae powder with mixture of β-carotene, zeaxanthin, cryptoxanthin and lutein	Australia	planktonaustralia.com
Nature Beta Technologies (NBT) Ltd.	β-carotene and 9-*cis*-β-carotene	Israel	nikken-miho.com

Sources: adapted from Mesquita et al. [85]. The website accessed on 26 June 2021.

**Table 4:** Summarizing of factors affecting biotechnological production of carotenoids.

Factor	Micro-Organism	Tested Condition	Main Finding	Reference
**pH**	*Rodothorula glutinis*	pH 5.91	Production of the highest total carotenoid contents and biomass production	[5]
	*Rodothorula glutinis*	pH 6.2	The highest content of total carotenoids was observed after 5 days of growth.	[35]
	*Rodothorula glutinis*	pH ≈ 4.0	High total carotenoid contents in a medium containing imidazole as an inducer of lycopene production.	[86]
	*Rodothorula glutinis*	pH 5.0	Production of the highest total carotenoid contents and biomass production	[77]
	*Rodothorula mucilaginosa*	pH 5.0	Increase in the production of carotenoid and biomass	[87]
	*Rodothorula acheniorum*	pH 5.85	Resulted in a 4.62-fold increase in accumulation of β-carotene	[88]
	*Sporidiobolus salmonicolor*	pH 4.0	Maximum concentration of total carotenoids	[42]
	*Rhodosporidium diobovatum*	pH 5.0	Production of the highest total carotenoid contents	[89]
	*Rodothorula rubra*	pH 5.0	Production of the maximum pigment and cell concentration	[61]
	*Rodothorula rubra*	pH 5.0	Optimum yeast growth, but also observed that torularhodin production was favored in the pH 6.0–7.0	[90]
	*Rhodotorula mucilaginosa and Rhodotorula toruloides*	pH 5.0	*R. mucilaginosa* produced the lowest amount of total carotenoids, while *R. toruloides* produced the highest total carotenoid	[91]
**Temperature**	*Rodothorula glutinis*	15 and 25 °C	The optimum temperature to produce carotenoids was 15 °C, while cell growth at 25 °C.	[35]
	*Rodothorula glutinis*	30 °C	The highest production of biomass and carotenoids	[37]
		25–30 °C	The production rate of carotenoids increased at temperatures above 30 °C	[6]
	*Rodothorula mucilaginosa*	25–30 °C	Increase in the carotenoid production rate; however, at temperatures above 30 °C, carotenoid biosynthesis appeared to be reduced	[52]
	*Rodothorula mucilaginosa*	22–34 °C	Maximum biomass production was obtained at 34 °C (pH 5.0), while maximum carotenoid synthesis was observed at 22 °C (pH 7.0)	[92]
	*Rodothorula mucilaginosa*	10–30 °C	Both biomass yield and carotenoid contents increased with temperature increase	[87]
	*Rodothorula acheniorum*	23 °C	Maximum optimized production of β-carotene	[88]
	*Rodothorula rubra*	20–30 °C	None significant difference for both production of carotenoids and biomass growth	[90]
	*Rodothorula diobovatum*	30 °C	Maximum cell growth	[89]
	*Sporidiobolus salmonicolor*	25 °C	The highest production of carotenoid	[93]
**Agitation and aeration rate**	*Rodothorula glutinis*	100 to 150 rpm	Lower cell growth due to the reduction of the availability of nutrients on the cell surface, while cell rupture was observed at > 250 rpm	[5]
	*Rodothorula glutinis*	100 rpm	The highest carotenoid contents after cultivation at 25 °C	[35]
	*Rodothorula glutinis*	125 rpm	Production of the highest total carotenoid concentrations	[86]
	*Rodothorula mucilaginosa*	150 rpm	Maximized carotenoid production after 72 h	[87]
	*Rhodosporidium toruloides*	160 rpm	The accumulation of carotenoids increased over time, reaching the maximum after 96 h of fermentation	[94]
	*Sporidiobolus salmonicolor*	1.5 vvm and 180 rpm	Maximized the production of carotenoids	[42]
	*Sporidiobolus pararoseus*	1.2 vvm and 158 rpm	Production of carotenoids was 3.5-fold higher in a stirred tank than agitation in shaking flasks (100–200 rpm)	[95]
**Light irradiation**	*Rodothorula glutinis*	weak white light irradiation	Inhibited cell growth; however, simultaneously, it showed a substantial increase in torularhodin production	[80]
	*Rodothorula glutinis*	light-emitting diodes (LEDs)	Carotenoid production increased when the yeasts were subjected to illumination by three LED lamps (800 mol/m^2^s)	[37]
